# *Ishige okamurae* Attenuates Neuroinflammation and Cognitive Deficits in Mice Intracerebroventricularly Injected with LPS via Regulating TLR-4/MyD88-Dependent Pathways

**DOI:** 10.3390/antiox12010078

**Published:** 2022-12-29

**Authors:** Oh-Yun Kwon, Seung-Ho Lee

**Affiliations:** Department of Nano-Bioengineering, Incheon National University, 119 Academy-ro, Incheon 22012, Republic of Korea

**Keywords:** *Ishige okamurae*, lipopolysaccharide, neuroinflammation, cognitive deficits, TLR-4, MyD88

## Abstract

Neuroinflammation is one of the critical causes of neuronal loss and cognitive impairment. We aimed to evaluate the anti-neuroinflammatory properties of *Ishige okamuae* using mice intracerebroventricularly injected with lipopolysaccharides (LPS) and LPS-treated C6 glioma cells. We found that the short- and long-term memory deficits of LPS-injected mice were improved by oral administration of *Ishige okamurae* extracts (IOE). LPS-induced neuronal loss, increase in amyloid-β plaque, and expression of COX-2 and iNOS were restored by IOE. In addition, LPS-induced activation of Toll-like receptor-4 (TLR-4) and its downstream molecules, such as MyD88, NFκB, and mitogen-activated protein kinases (MAPKs), were significantly attenuated in the brains of mice fed with IOE. We found that pretreatment of IOE to C6 glioma cells ameliorated LPS-induced expression of TLR-4 and its inflammatory cascades, such as MyD88 expression, reactive oxygen species production, MAPKs phosphorylation, and NFκB phosphorylation with consequent downregulation of COX-2, iNOS, proinflammatory cytokines, and nitric oxide expression. Furthermore, IOE (0.2 µg/mL) was found to have equivalent efficacy with 10 μM of MyD88 inhibitor in preventing LPS-induced inflammatory responses in C6 glioma cells. Taken together, these results strongly suggest that IOE could be developed as a promising anti-neuroinflammatory agent which is able to control the TLR-4/MyD88-dependent signaling pathways.

## 1. Introduction

Alzheimer’s disease is a representative neurodegenerative disease that induces neuronal loss and cognitive deficits [1]. To prevent the progress of AD, many studies have focused on finding the central mediators of AD pathogenesis, and as a result, amyloid-β (Aβ) peptide and tau protein have been suggested as primary causes of AD [2]. However, there are increasing demands for discovering the new central mediator that can control AD progress, since the anti-AD drugs targeting Aβ and tau proteins are not showing satisfactory results in clinical trials [3,4]. Recently, neuroinflammation has received much attention as a key regulator in AD progress because it has been reported to be closely related to synapse loss and cognitive impairment [5]. Many studies have suggested that systemic inflammation, such as infection, sepsis, and periodontitis, leads to the activation of glial cells, resulting in increased reactive oxygen species (ROS) and circulating pro-inflammatory cytokines that contribute to AD progress [6,7,8]. Gram-negative bacteria, which can provoke systemic inflammation, are able to reach the brain through the blood–brain barrier (BBB) [9], and lipopolysaccharides (LPS), an essential cell wall component of gram-negative bacteria, are also known to penetrate the BBB [10].

LPS is a potential endotoxin that stimulates immune cells to induce the production of proinflammatory cytokines. It has been reported that the concentration of LPS in the blood of AD patients is higher than that of normal people, and 3–6 times more bacteria is detected in the brains of AD patients than that of healthy people [11,12]. LPS-induced systemic inflammation in animal models induces neuroinflammation with neuronal loss and cognitive impairments [13,14]. Thus, LPS-mediated proinflammatory progress has been recognized as an upstream pathologic event that can trigger AD pathology. 

The neuroinflammatory response by LPS is known to be initiated through the activation of Toll-like receptor-4 (TLR-4) and its downstream signaling molecules, such as the myeloid differentiation primary response 88 (MyD88), reactive oxygen species (ROS), mitogen-activated protein kinases (MAPKs), and nuclear factor kappa-light-chain-enhancer of activated B cells (NFκB) [15,16]. TLR-4 activation induced by LPS triggers the expression of proinflammatory mediators, such as cyclooxygenase-2 (COX-2), nitric oxide synthase (iNOS), and proinflammatory cytokines, which are considered harmful mediators in neurodegeneration and cognitive deficits [14]. Since most TLR-4 mediated inflammatory responses are initiated by recruiting MyD88, a key intracellular adaptor protein of TLR-4 [17], TLR-4/MyD88 dependent pathway is a potential target for curing the AD progress induced by LPS-mediated neuroinflammation.

*Ishige okamurae* is a brown algae that is easily found in East Asia [18]. *Ishige okamurae* is an edible seaweed and called ‘pae’ in South Korea. Brown algae including *Ishige okamurae* are known to contain various bioactive components such as phlorotannins and polysaccharide [19]. Actually, many studies have shown several biological properties of *Ishige okamurae*, such as its anti-inflammatory and hypoglycemic activities [20,21]. In addition, anti-glycation and renal protective activity of *Ishige okamurae* were recently reported by Kim M et al. [22]. In our previous studies, we demonstrated that *Ishige okamurae* extracts (IOE) have protective roles against Aβ (Aβ_25–35_) and trimetyltin (TMT)-induced cognitive deficits and neuronal loss in the mouse brain [23,24]. We also proved that IOE plays a neuroprotective role in glutamate-induced exitotoxicity [24]. However, the effects of IOE on LPS-induced neuroinflammation and its underlying molecular mechanisms remain unclear. Herein, we elucidated the effects of IOE on neuroinflammation-induced cognitive impairments and neuronal loss in a mouse model of intracerebroventricular (ICV) LPS injection, and those effects were further evaluated in LPS-treated C6 glioma cells. Furthermore, we focused on describing the molecular mechanisms by which IOE modulates LPS-induced neuroinflammatory processes.

## 2. Materials and Methods

### 2.1. Materials

We used *Ishige okamurae* extract (IOE), which was prepared as described in our previous study [23]. Briefly, *I. okamuare*, which was harvested on Jeju Island in Republic of Korea, was ground, powdered, and then extracted with 70% ethyl alcohol. The extracts were concentrated and lyophilized using an evaporator system (Heidolph Instruments GmbH and Co., Schwabach, Germany) and a freeze-drying system (ilShin BioBase, Seoul, Republic of Korea), respectively. LPS from *Escherichia coli* O111:B4 was obtained from Sigma Aldrich (Louis, MO, USA). Rabbit anti-phospho-ERK (9102S), rabbit anti-ERK (9101S), rabbit anti-phospho-p38 (9212S), rabbit anti-p38 (9211S), rabbit anti-phospho-JNK (9251S), rabbit anti-JNK (9252S), mouse anti-NFκB (6956S), rabbit anti-phospho-NFκB (3031S), rabbit anti-Bax (2772S), rabbit anti-Bcl2 (2876S), rabbit anti-cleaved caspase 3 (9664S), rabbit anti-MyD88 (4283S), rabbit anti-COX-2 (12282S) antibody are purchased from Cell Signaling (Danvers, MA, USA). Rabbit anti-iNOS (NB 300–605) was purchased from Novus (Centennial, CO, USA), and rabbit anti-TLR-4 (48–2300) was obtained from Invitrogen Co. (Middlesex County, MA, USA). Myeloid differentiation factor 88 (MyD88) inhibitor (NBP2–29328) was purchased from NOVUS Biologicals (Boston, MA, USA).

### 2.2. Animal Experiments

C57BL/6 male mice (9 weeks old) were purchased from NARA Biotech (Seoul, South Korea). The mice were raised in a room maintained of a 12-h light/12-h dark cycle, humidity (55 ± 10%), and temperature (22 ± 3 °C) with free access to food (Orient Bio, Seoul, South Korea) and water ad libitum. After one week of adaptation, the mice were distributed into four groups: control group (phosphate buffered saline (PBS) injection and oral PBS gavage at 100 μL, *n* = 7), IOE group (PBS injection and oral IOE gavage at 20 mg/kg bw/day, *n* = 7), LPS group (LPS injection at oral PBS gavage at 100 μL, *n* = 7), and LPS **+** IOE group (LPS injection and oral IOE gavage at 20 mg/kg bw/day). LPS was prepared by dissolving it in distilled water, and 3 µL (4 µg/µL) of LPS was injected into the ICV region of mouse brains once after ingestion of IOE for 28 days, according to the methods of Kim H.Y. et al. [25]. After LPS or PBS ICV injection, IOE was further administered to mice for 1 week, and then the Y-maze test and Morris water maze teste were performed. All experiments were performed in accordance with the guidelines for the management and use of laboratory animals at Incheon National University and approved by the Incheon National University Institutional Animal Care and Use Committee (INU-ANIM2019–17).

### 2.3. Y-Maze Test

The short-term memory deficit of each mouse group was estimated using the Y-maze test, according to the methods of Kwon O.Y. et al. [23,24]. We used a Y-maze device consisting of three arms (36 cm long, 15 cm high, 3 cm bottom width, and 9 cm top width) marked A, B, or C. After placing the mouse at the center, the movement of each mouse was recorded for 8 min using a SMART 3.0 video-tracking system (Harvard Apparatus, Holliston, MA, USA). Actual alteration was estimated when a mouse first entered A, then B, then C; however, when a mouse entered A, then B, then A did not count as an alternation. Possible alterations were calculated as the total number of arm entries-2. The rate of alternation behavior was calculated as: alternations behavior (%) = (actual alternations)/(possible alternations) × 100.

### 2.4. Morris Water Maze Test

The spatial and long-term memory deficits of each mouse group were estimated using the Morris water maze (MWM) test according to the methods of Kwon OY et al. [23,24]. For the MWM test, we used a circular pool (60 cm in height and 90 cm in diameter) filled with water (22 ± 2 °C) and white ink (Wilton Industries, Inc., Woodridge, IL, USA) to make the color of the water white. The circular pool was divided into four equal quadrants (E, W, S, and N), and a platform (30 cm in height and 6 cm in diameter) was placed in the center of the W zone. The movement of the mice in the pool was recorded for 60 s. In a training session (8 days after LPS injection), the mice were allowed to swim to escape three times a day. The probe test (13 days after LPS injection) was performed for 60 s without a platform, and the time spent in the W zone was estimated using a SMART 3.0 video-tracking system (Harvard Apparatus, Holliston, MA, USA).

### 2.5. Tissue Staining

Brain tissue was fixed and preserved in formaldehyde solution (10% *w*/*v*). Tissue sections (5 μm) were deparaffinized twice in xylene solution (100%, *w*/*v*) (Daejung, Gyeonggi, Republic of Korea) for 5 min, then mounted with 100% ethanol (Merck, Darmstadt, Germany) for 10 min, 95% ethanol for 5 min, and 70% ethanol for 1 min. The slides were then rinsed twice with water. For hematoxylin and eosin staining, slides were first stained with hematoxylin solution (Sigma-Aldrich, St. Louis, MO, USA) for 8 min and then stained with eosin solution (25% *w*/*v*) (Sigma-Aldrich, St. Louis, MO, USA) for 1 min. Slides were finally fixed with 100% xylene for 3 min, and then the nuclear clumping-positive cells and the number of viable cells in the hippocampus region were counted under a microscope (Nikon, Tokyo, Japan). For thioflavin S staining, the slides were first stained with propidium iodide (PI) solution (5 µg/mL, ACROS Organics, Hampton, NH, USA) at room temperature for 15 min. After washing twice with 80% ethanol, the slides were immersed in thioflavin S solution (1% *w*/*v*, Sigma-Aldrich, St. Louis, MO, USA) at room temperature for 15 min. After washing with 80% ethanol once and water twice, the positive signals of PI (red) and thioflavin S (green) were observed using a confocal laser scanning microscope (Nikon, Tokyo, Japan). Plaque burden (plaque area/number of plaques) was estimated using the Image J analysis program.

### 2.6. Cell Culture and Cell Viability Assay

C6 glioma cells were purchased from the American Type Culture Collection (ATCC) and cultured in high-glucose Dulbecco’s modified Eagle medium (DMEM) (HyClone, Logan, UT, USA) contained with fetal bovine serum (10% *v*/*v*, Corning, NY, USA) and streptomycin/penicillin (1% *w*/*v*, Sigma-Aldrich, St. Louis, MO, USA) at 37 °C in a CO_2_ incubator. For the cell viability assay, C6 glioma cells (5 *×* 10^3^ cells/well) were seeded in a 96-well plate and cultured for 24 h. IOE (0–2 mg/mL) was then treated with C6 glioma cells and further incubated for 24 h. The 4-[3-(4-Iodophenyl)-2-(4-nitrophenyl)-2H-5-tetrazolio]-1,3-benzene disulfonate (WST-1) reagent was then added to each well, and the absorbance was measured at 450 nm according to the manufacturer’s protocol (Dozen, Seoul, Republic of Korea).

### 2.7. Nitric Oxide Assay

C6 glioma cells (5 × 10^3^ cells/well) were seeded in a 96-well plate and cultured for 24 h in a CO_2_ incubator. IOE was pretreated for 2 h, and then LPS (1 μg/mL) or PBS was added and further incubated for 15 h. The supernatant (50 μL) of each well was reacted with 50 μL of Griess reagent (iNtRON, Gyeonggi, Republic of Korea) at room temperature for 20 min, and the absorbance was measured at 540 nm using a microplate reader (Bio-Rad, CA, USA).

### 2.8. Reactive Oxygen Species Measurements

C6 glioma cells (5 × 10^3^ cells/well) were seeded on a black plate (96-well, SPL Life Sciences, Gyeonggi, Republic of Korea) and cultured for 24 h in a CO_2_ incubator. The cells were treated with LPS (1 μg/mL), MyD88 inhibitor (10 μM) (NBP2–29328), or IOE and further incubated for 16 h in a CO_2_ incubator. After washing twice with PBS, 50 μM of 2–7dichlorofluorescin (DCFH-DA) solution (Sigma-Aldrich Co., St. Louis, MO, USA) was added, and the sample was further incubated for 1 h in a CO_2_ incubator. The samples were washed with PBS, and 100 μL of PBS was added to each well. Fluorescence intensity was measured at excitation 485 nm and emission 535 nm using a microplate fluorometer (Tecan, Mannedorf, Switzerland).

### 2.9. Real-Time Polymerase Chain Reaction

C6 glioma cells (10^5^ cells/well) were cultured in 6-well plates for 24 h. After treatment with IOE, LPS, or MyD88 inhibitor (NBP2–29328) for 15 h, total RNA was extracted using TRIzol reagent (Invitrogen, Waltham, MA, USA). Complementary DNA (cDNA) was synthesized with 1.5 μg of total RNA and 10 pM of oligo dT primers, and quantitative real-time polymerase chain reaction (qRT-PCR) was performed using SYBR Green Realtime PCR Master Mix (Toyobo Co., Tokyo, Japan) in a CFX Connect Real-Time PCR detection system (Bio-Rad Co., Hercules, CA, USA). The expression of each gene was normalized with that of glyceraldehyde 3-phosphate dehydrogenase (GAPDH) and determined using the comparative Ct method. The sequences of the cyclooxygenase-2 (COX-2) primers were 5′-TGCTGCCGGACACCTTCA-3′ (forward) and 5′-AACCCGGCCAGCAATCTG-3′ (reverse), while those of the inducible nitric oxide synthase (iNOS) primers were 5′-AGGAGGCCGCATGACCTT-3′ (forward) and 5′-TTGGGTTTTCCGGGCAGC-3′ (reverse), and those of the GAPDH primers were 5′-ACGGGAAGCTCACTGGCA- 3′ (forward) and 5′-TCCAGGCGGCATGTCAGA-3′ (reverse), tumor necrosis factor- α (TNF-α) primers were 5′-TCCAGAACTCCAGGCGGT-3′ (forward) and 5′-TGGGCTCATACCAGGGCT-3′ (reverse), interleukin-6 (IL-6) primers were 5′-TCTCGAGCCCACCAGGAA-3′ (forward) and 5′-GGCAACTGGCTGGAAGTCT-3′ (reverse), Interleukin-1β (IL-1β) primers were 5′-AAATCCCTGTGGCCTGGG- 3′ (forward), and 5′-GTGGGTGTGCCGTCTTTC-3′ (reverse). The primers of COX-2, iNOS, and GAPDH were designed in our previous study [23] and TNF-α, IL-6, and IL-1β primers were newly designed in this study.

### 2.10. Western Blot Analysis

Treated C6 glioma cells were washed once with ice-cold PBS and then harvested with cell lysis buffer (70 μM ethylene-diamine-tetra-acetic acid (EDTA), 20 mM Tris-HCl (pH 7.4), and Nonidet P-40 (1%, *w*/*v*), 150 mM NaCl, 1X phosphatase inhibitor cocktail (Cell Signaling, Danvers, MA, USA)). After incubating on ice for 1 h, the cell lysates were centrifuged (13,000× *g*) at 4 °C for 10 min. The supernatant was isolated and used to measure the protein concentration. Mouse brain tissue (10 mg) was immersed in cell lysis buffer (70 μM ethylene-diamine-tetra-acetic acid (EDTA), 20 mM Tris-HCl (pH 7.4), and Nonidet P-40 (1%, *w*/*v*), 150 mM NaCl, 1X phosphatase inhibitor cocktail (Cell Signaling, Danvers, MA, USA)) and homogenized using a homogenizer (Huanyu Instrument, Zhejiang, China). After centrifuging (13,000× *g*) at 4 °C for 10 min, the supernatant was separated and used to estimate protein concentration. Protein samples (30 μg) were separated in sodium dodecyl sulfate-polyacrylamide polyacrylamide gel electrophoresis (SDS-PAGE). The gel was transferred to a nitrocellulose membrane and incubated with nonfat milk (5%, *w*/*v*) at 4 °C for 15 h. The membrane was then incubated with each primary antibody (1:1500) at 4 °C for 12 h and washed with washing buffer (20 mM Tris, 136 mM NaCl, Tween 20 (0.4%, *w*/*v*), pH 7.4) three times. Horseradish peroxidase (HRP)-conjugated rabbit secondary antibody (1:3000) (Abcam, Cambridge, UK) reacted with membrane at room temperature for 2 h. The expression of each band was visualized with an enhanced chemiluminescence (ECL) detection reagent (Bio-Rad, Hercules, CA, USA).

### 2.11. Statistical Analysis

Data were expressed as mean ± standard deviation (SD) or standard error of the mean (SEM). Two-way ANOVA, followed by Tukey’s post hoc multiple comparisons test, was performed to estimate statistical differences between groups using a GraphPad Prism 5 (GraphPad Software Inc., La Jolla, CA, USA). A value of *p* < 0.05 was considered to be statistically significant.

## 3. Results

### 3.1. Oral Administration of Ishige okamurae Extract (IOE) Attenuated LPS-Induced Cognitive Deficits

**Figure 1:**
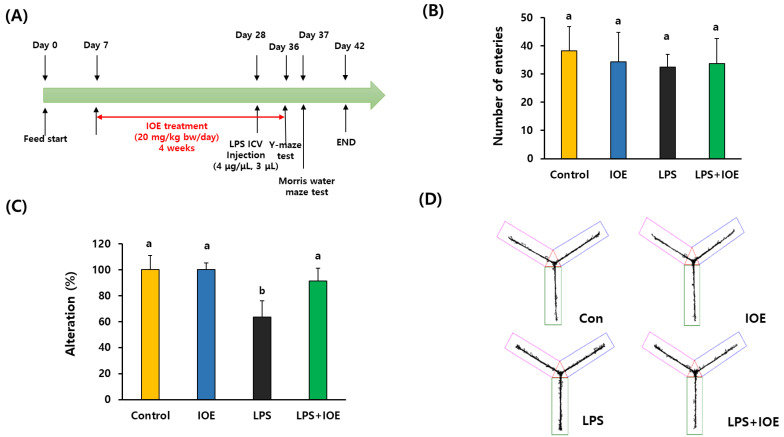
IOE attenuated the LPS-mediated spatial memory impairment in mice. IOE was orally administered to mice for 4 weeks and then LPS was ICV injected to mice (*n* = 7) (**A**). LPS-induced cognitive impairment was investigated by using a Y-maze test. The number of entries (**B**) alteration (%) (**C**) and path tracing (**D**) of each mice group were estimated by using a SMART 3.0 video tracking system. Each data value is expressed as the mean ± SD. Different letters (a,b) indicate the significant differences between groups (*p* < 0.05). Each value is assigned as a and b sequentially from the highest value.

To investigate the effect of IOE on cognitive deficits in the LPS-induced neuroinflammatory mouse model, IOE was orally administrated to mice for 28 days, and then LPS was intracerebroventricularly injected into the mouse brain (Figure 1A). One week after injection, the Y-maze and Morris water maze tests were started to estimate learning and memory impairments. As shown in Figure 1B,C. The number of entries among the mouse groups was not significantly different, but spontaneous alteration (%) of the LPS mouse group (63.52 ± 12.43%) was significantly (*p* < 0.05) decreased when compared with that of the control (100 ± 10.86%) and IOE mouse groups (101.98 ± 4.84%). However, it was significantly restored in the LPS *+* IOE mouse group (91.20 ± 9.42%). In addition, we found that the LPS mouse group showed imbalanced movement when compared with the control and IOE mouse groups. However, the LPS *+* IOE mouse group exhibited a similar movement route to that of the control and IOE mouse groups (Figure 1D). These results indicate that the abnormal spatial working memory induced by ICV LPS injection could be attenuated by the oral administration of IOE.

**Figure 2:**
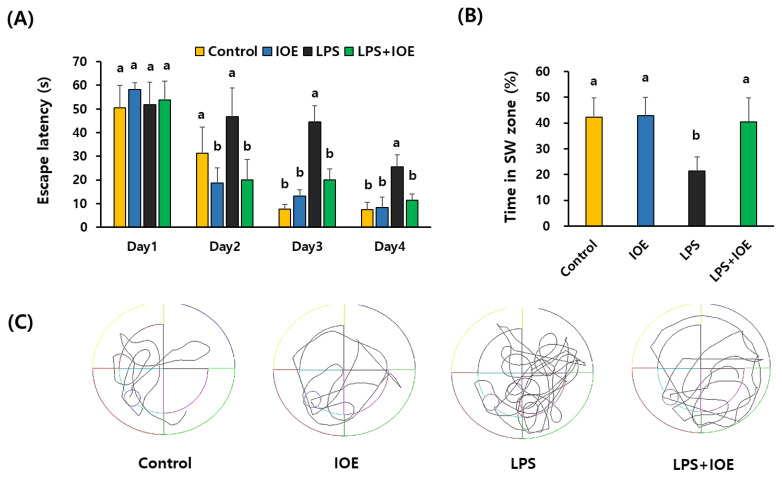
LPS-induced loss of long-term memory in mice was ameliorated by IOE. Long-term memory impairments of each mice group were estimated by using Morris water maze test. Escape latency (s) (**A**), time to stay in W zone at day 5 (%) (**B**), and movement patterns during probe test (**C**) of each mice group was estimated (*n* = 7). Data shown are the mean ± SD. Different letters (a,b) indicate the significant differences between groups (*p* < 0.05). Each value is assigned as a and b sequentially from the highest value.

The effects of oral administration of IOE on the long-term learning and spatial memory of mice with intracerebroventricular LPS injection were assessed using an MWM test. As shown in Figure 2A, the time to reach the platform (escape latency) was gradually decreased during the MWM tests in all mouse groups, indicating that the memory about the location of the escape zone in all mouse groups was gradually increased during the experimental period (4 days). However, we found that the escape latency of the LPS mouse group (44.55 ± 6.91 s) was significantly higher than that of the control (7.74 ± 1.99 s) and IOE mouse groups (13.13 ± 2.73 s) from day 3 of the MWM test. Furthermore, we found that the LPS mouse groups spent significantly (*p* < 0.05) less time (21.41 ± 5.44%) in the W zone where the escape platform existed than the control (42.32 ± 7.46%) and IOE mouse groups (42.85 ± 7.07 s). However, the time spent by the LPS *+* IOE mouse group in the W zone (40.50 ± 9.28%) was significantly recovered (*p* < 0.05) to an extent similar to that of the control and IOE mouse groups (Figure 2B). In addition, the distance to reach the W zone of the LPS *+* IOE mouse group was reduced compared to the LPS mouse group. (Figure 2C). These results suggest that LPS-induced cognitive impairments, such as learning and memory deficits, were ameliorated by IOE administration. 

### 3.2. LPS-Induced Neuronal Loss and Neuroinflammation in Mouse Brains Were Attenuated by IOE Administration

**Figure 3:**
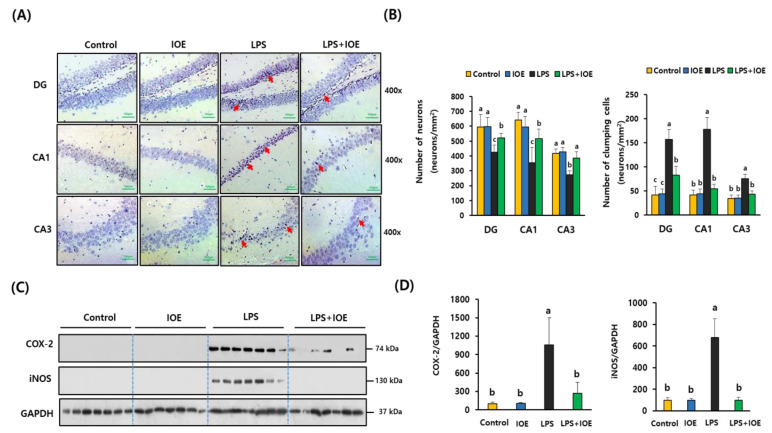
LPS-induced neuronal loss and expression of inflammatory mediators in mice brain were inhibited by oral administration of IOE. DG, CA1 and CA3 region in mice brain of each mice group were visualized by hematoxylin–eosin (H&E) staining (*n* = 7) (**A**). The number of viable cells and clumping cells which has pyknotic nuclei in each region was counted and tabulated (**B**). The expression of COX-2 and iNOS in mice brain of each mouse group was evaluated by western blotting (*n* = 7) (**C**) and the density of each band was estimated by using image J program and tabulated (**D**). Red arrows indicate the pyknotic nuclei. Magnification 400×. Scale bar = 100 μm. Data shown are the mean ± SD and different letters (a–c) indicate the significant differences between groups (*p* < 0.05). Each value is assigned as a–c sequentially from the highest value.

After confirming the effects of IOE on LPS-induced cognitive impairments, the brain tissues of each mouse group were isolated and stained with hematoxylin–eosin solution to evaluate the effects of IOE on LPS-mediated neuronal loss. As shown in Figure 3, the number of surviving neurons in the dentate gyrus (DG), CA1, and CA3 region of the hippocampus was significantly decreased (*p* < 0.05) in the LPS mouse group (DG: 425.71 ± 46.03, CA1: 354.28 ± 102.39, CA3: 274.28 ± 25.40) compared with that of the control (DG: 595 ± 84.68, CA1: 640.42 ± 52.159, CA3: 417.85 ± 27.90) and IOE mouse groups (DG: 595 ± 84.68, CA1: 640.42 ± 52.159, CA3: 417.85 ± 27.90). Furthermore, the number of pyknotic nuclei, which is easily detected in cells undergoing apoptosis or necrosis, was significantly (*p* < 0.05) increased in the hippocampus region of the LPS mouse group, but these neuronal damages were significantly ameliorated (*p* < 0.05) in the hippocampus region of LPS *+* IOE mice (DG: 82.57 ± 18.45, CA1: 54 ± 9.81, CA3: 42.57 ± 6.87). These results suggest that oral administration of IOE efficiently attenuated LPS-induced neuronal loss in the hippocampus region of mouse brains.

**Figure 4:**
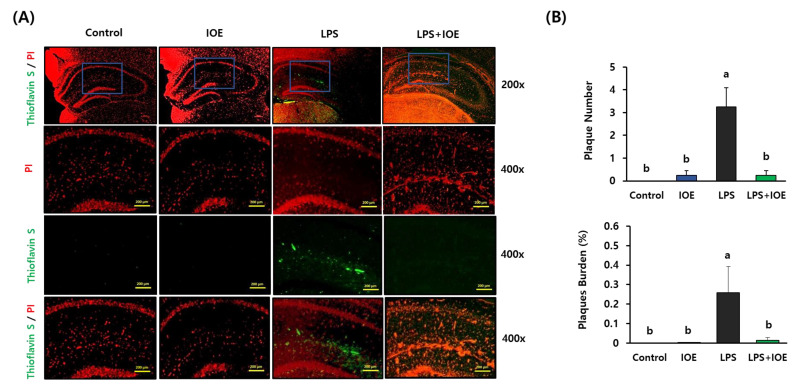
Oral administration of IOE attenuated the LPS-induced Aβ plaque formation in mice brain. The Hippocampal region of each mice group was stained with propidium iodide (PI) and Thioflavin S (green) (*n* = 7) (**A**). The number of plaque and a percentage of plaque burden (plaque area/number of plaques) was calculated with the Image J program (**B**). Magnification 200× and 400×. Scale bar = 50 µm. Data shown are the mean ± SD and different letters (a,b) indicate the significant differences between groups (*p* < 0.05). Each value is assigned as a and b sequentially from the highest value.

Since LPS is a potent stimulator in inflammation reactions, several inflammation-related proteins, such as COX-2 and iNOS, could be increased in the brain tissues of the LPS-injected mouse groups. As expected, significant (*p* < 0.05) upregulation of COX-2 and iNOS expression was detected in the brain of the LPS mouse group compared with that of the control and IOE mouse groups, indicating that neuroinflammatory progress was increased in brain tissues of the LPS mouse group. However, the expression of COX-2 and iNOS was reduced in the brain tissues of the LPS *+* IOE mouse group to levels similar to those of the control and IOE mouse groups (Figure 4C,D). These results suggest that the oral administration of IOE hindered the inflammatory responses in the brain tissue induced by ICV injection of LPS.

### 3.3. IOE Administration Attenuated the LPS-Induced Aβ Plaque Formation in Mouse Brains

To investigate the effects of IOE on the LPS-mediated Aβ plaque formation in the mouse brain, Aβ plaque positive area in the hippocampus region was visualized with thioflavin S staining (Figure 4). We found that the number of plaques and the percentage of plaque burden increased in the brains of the LPS mouse group compared with that of the control and IOE mouse groups, but it was significantly abolished in the LPS + IOE mouse group. These results suggest that the oral administration of IOE could be effective in diminishing the LPS induced Aβ plaque formation which has been considered a critical cause of neuronal loss and cognitive impairments in AD.

### 3.4. Activation of TLR-4 and its Downstream Signaling Pathway in LPS-Injected Mouse Brains Was Downregulated by Oral Administration of IOE

**Figure 5:**
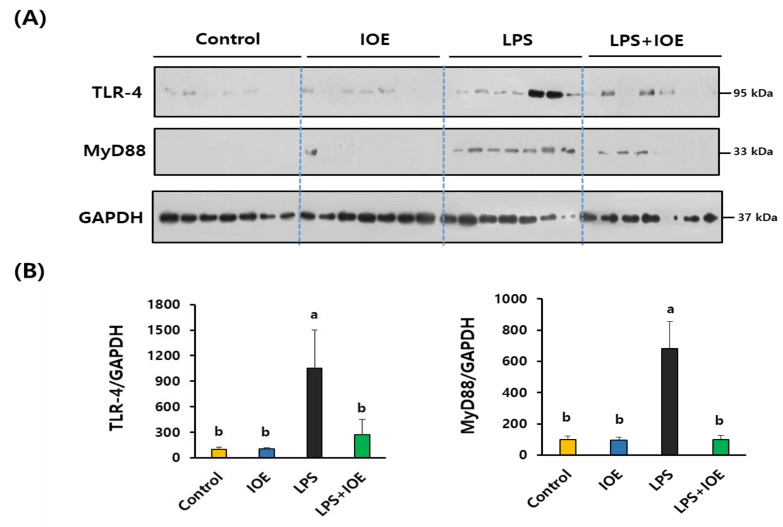
The expression of TLR-4 and MyD88 in brain tissue of LPS-injected mice was attenuated by oral administration of IOE. Total protein was extracted from brain tissues of each mice group and used for estimating the expression of TLR-4 and MyD88 through western blotting (*n* = 7) (**A**). The density of each band was calculated by using Image J program and tabulated (**B**). Data shown are the mean ± SD and different letters (a,b) indicate the significant differences between groups (*p* < 0.05). Each value is assigned as a and b sequentially from the highest value.

**Figure 6:**
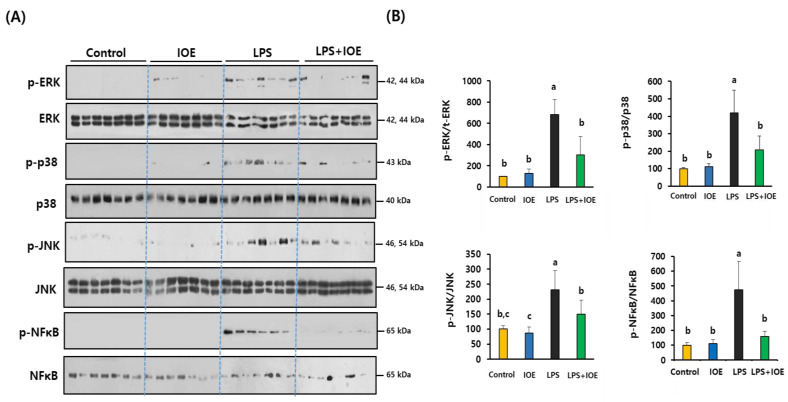
Abnormal phosphorylation of MAPKs and NFκB in brain tissue of LPS-injected mice was ameliorated by IOE administration. The expression of phosphorylated MAPKs (ERK, p38 and JNK), total MPAKs phosphorylated NFκB and total NFκB was evaluated by using western blotting (*n* = 7) (**A**). The density of each band was calculated by using Image J program and tabulated (**B**). Data shown are the mean ± SD and different letters (a–c) indicate the significant differences between groups (*p* < 0.05). Each value is assigned as a, b and c sequentially from the highest value.

To determine the molecular mechanisms by which IOE attenuated LPS-induced neuroinflammatory responses in the brain, we investigated the activation of TLR-4, which plays a critical role in transmitting LPS-induced inflammatory signals. As shown in Figure 5, ICV injection of LPS dramatically induced the expression of TLR-4 (859.07 ± 449.05%), and its adaptor protein MyD88 (680.49 ± 174.83%) indicated that TLR-4 was activated in the brains of LPS-injected mice. Intriguingly, these expressions were significantly decreased in the LPS *+* IOE mouse group (TLR-4: 272.52 ± 176.42%, MyD88: 99.41 ± 25.95%), suggesting that oral administration of IOE hindered LPS-induced TLR-4 activation in the mouse brain. We also found that the phosphorylation of NFκB and MAPKs (ERK, p38, and JNK), which are downstream signaling pathways of TLR-4, was attenuated in the brains of the IOE mouse group when compared with that of the LPS mouse group (Figure 6). Taken together, these results suggest that the oral administration of IOE effectively ameliorated LPS-induced TLR-4-mediated intracellular signaling pathways in mouse brains. 

### 3.5. IOE Attenuated LPS-Induced TLR-4 Activation in C6 Glioma Cells

**Figure 7:**
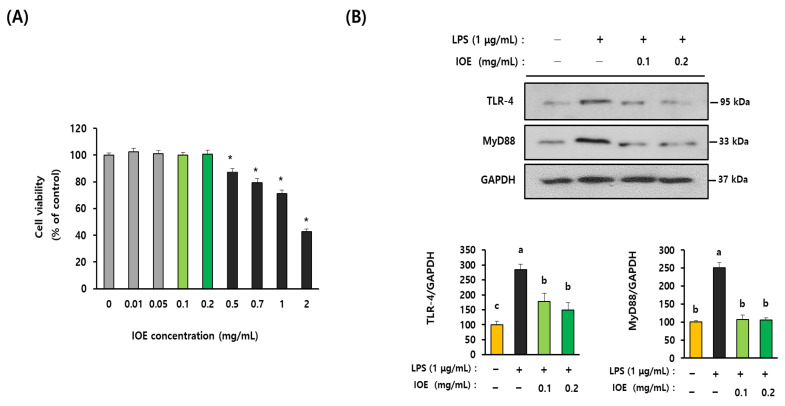
IOE attenuated the expression of TLR-4 and MyD88 in LPS-treated C6 glioma cells. Cytotoxicity of IOE on the C6 glioma cells was estimated by using a WST-1 cell viability assay kit (**A**). The expression of TLR-4 and MyD88 on the C6 glioma cells treated with LPS was evaluated by western blotting. The density of each band was calculated by using Image J program and tabulated (**B**). These experiments were conducted at least three times with similar results. Data shown are the mean ± SD and different letters (a–c) indicate the significant differences among treatments (*p* < 0.05). Each value is assigned as a, b and c sequentially from the highest value. * indicates significant differences when compared to the control (non-treatment) *p* < 0.05.

**Figure 8:**
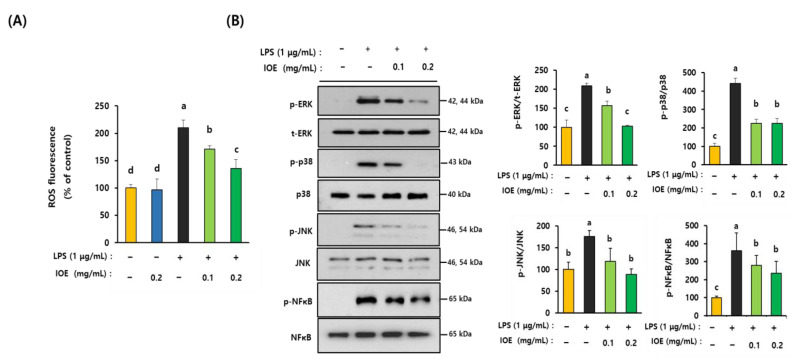
LPS-induced dysregulation of ROS, MAPKs and NFκB on C6 glioma cells were restored by pretreatment of IOE. C6 glioma cells were pretreated with IOE (0.1 and 0.2 mg/mL) and then stimulated with LPS (1 µg/mL). ROS production was evaluated by using a microplate fluorometer (**A**). The expression of each protein was estimated by western blotting. The density of each band was calculated by using Image J program and the ration of phosphorylated MAPKs/total MAPKs and phosphorylated NFκB/total NFκB was calculated and tabulated (**B**). These experiments were repeated three times with similar results. Data shown are the mean ± SD and different letters (a–c) indicate the significant differences among treatments (*p* < 0.05). Each value is assigned as a, b and c sequentially from the highest value.

To elucidate the detailed mechanisms by which IOE regulated LPS-mediated inflammatory responses, we investigated the effects of IOE on LPS-mediated inflammatory responses in C6 glioma cells. We found that IOE concentrations of up to 0.2 mg/mL did not have cytotoxic effects on the cells (Figure 7A). Based on these results, C6 glioma cells pretreated with nontoxic concentrations of IOE (0.1 and 0.2 mg/mL) were evaluated for the LPS-induced expression of TLR-4 and MyD88. As shown in Figure 7B, the expression of TLR-4 and MyD88 was increased when LPS was treated with C6 glioma cells, but it was significantly (*p* < 0.05) diminished in C6 glioma cells treated with non-toxicological levels of IOE (0.1 and 0.2 mg/mL). Treating C6 glioma cells upregulated ROS production, but it was dose dependently attenuated by IOE treatments (Figure 8A). Furthermore, the LPS-induced over-phosphorylation of MAPKs (ERK, p38, and JNK) and NFκB in C6 glioma cells was significantly (*p* < 0.05) decreased by IOE treatments (Figure 8B). These results suggest that LPS-induced TLR-4 intracellular signals, such as ROS NFκB and MAPKs, which play an important role in transmitting inflammatory responses, could be effectively attenuated by IOE treatments in C6 glioma cells.

### 3.6. LPS-Mediated Expression of Inflammation Mediators Was Inhibited by IOE Treatment in C6 Glioma Cells

**Figure 9:**
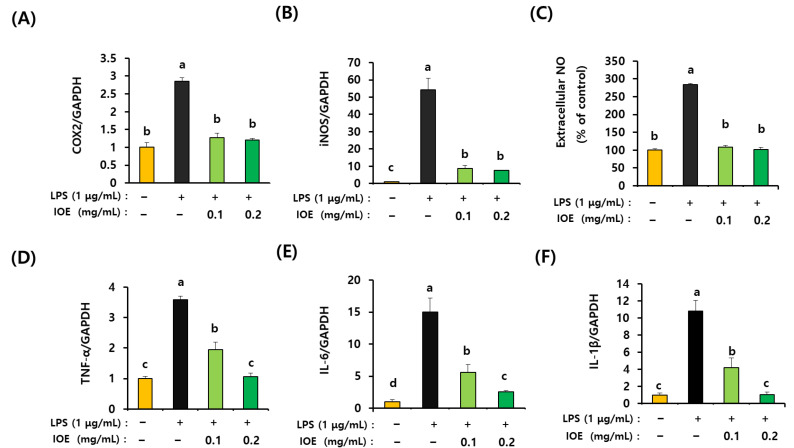
IOE ameliorated the inflammatory mediators in C6 glioma cells treated with LPS. C6 glioma cells was pretreated with non-toxicological levels of IOE (0.1 and 0.2 mg/mL) and then LPS (1 ug/mL) was treated to C6 glioma cells for 15 h. The expression of COX-2 (**A**), iNOS (**B**), TNF- α (**D**), IL-6 (**E**), and IL-1β (**F**) was estimated qRT-PCR and the amount of extracellular NO (**C**) in cell media was evaluated by using a microplate reader. These experiments were repeated three times with similar results. Data shown are the mean ± SD and different letters (a–d) indicate the significant differences among treatments (*p* < 0.05). Each value is assigned as a, b, c and d sequentially from the highest value.

Since the expression of inflammatory-related mediators, such as COX-2 and iNOS, was found to be downregulated in the brain tissue of the IOE mouse group when compared with that of the LPS mouse group (Figure 3C), we checked again the effects of IOE on the LPS-induced expression of COX-2 and iNOS on C6 glioma cells. As shown in Figure 9A,B, the increase in COX-2 and iNOS expression on the LPS-treated C6 glioma cells was inhibited by pretreatment with non-toxic levels of IOE. Further, the amount of extracellular nitric oxide (NO) produced by LPS administration was significantly (*p* < 0.05) decreased in IOE-treated C6 glioma cells (Figure 9C). The expression of proinflammatory cytokines such as TNF-α, IL-6 and IL-1β induced by LPS treatment was dose-dependently attenuated in IOE-treated C6 glioma cells (Figure 9D–F). Taken together, these results indicate that IOE effectively attenuated the upregulation of inflammatory mediators when C6 glioma cells were stimulated by LPS.

### 3.7. IOE Has Equivalent Efficacy with the MyD88 Inhibitor in the Inhibition of LPS-Mediated Inflammatory Responses

**Figure 10:**
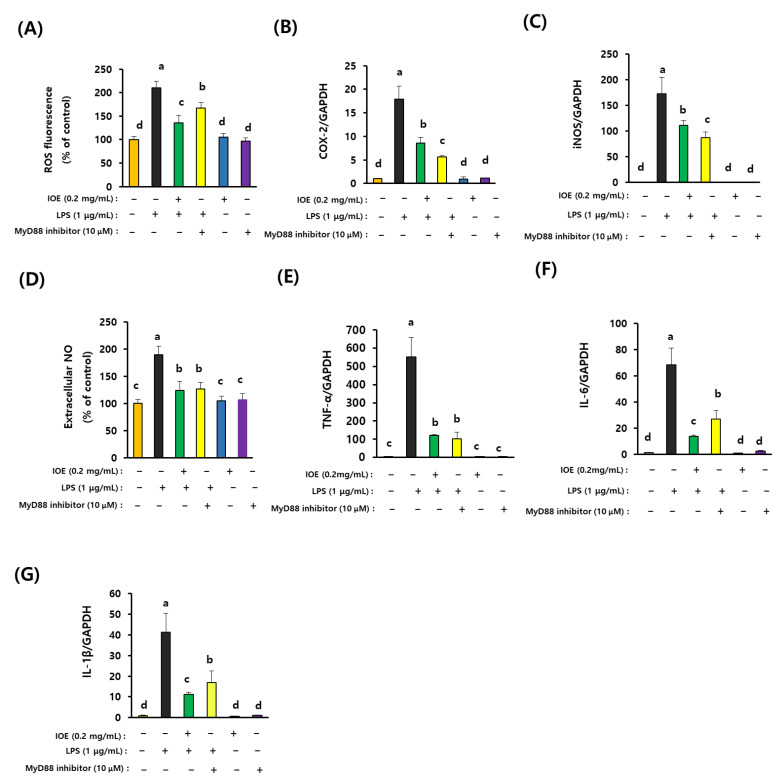
IOE showed equivalent anti-inflammatory activity with MyD88 inhibitor. The inhibitory activity of IOE (0.2 µg/mL) was compared with MyD88 inhibitor (10 µM) on the LPS-induced expression of inflammatory mediators such as ROS (**A**), COX-2 (**B**), iNOS (**C**), extracellular NO (**D**), TNF-α (**E**), IL-6 (**F**), and IL-1β (**G**) in C6 glioma cells. These experiments were repeated three times with similar results. Data shown are the mean ± SD and different letters (a–d) indicate the significant differences among treatments (*p* < 0.05). Each value is assigned as a, b, c and d sequentially from the highest value.

Lastly, we compared the inhibitory efficacy of IOE with that of the MyD88 inhibitor on LPS-induced inflammatory responses. As shown in Figure 10, LPS-induced ROS overproduction and the expression of COX-2, iNOS, extracellular NO, and proinflammatory cytokines were mostly ameliorated in C6 glioma cells pretreated with the MyD88 inhibitor (NBP2–29328), indicating that MyD88 plays a central role in the transmission of LPS-mediated inflammatory signals on C6 glioma cells. Interestingly, we found that pretreatment with IOE (0.2 mg/mL) diminished LPS-mediated inflammatory responses as much as MyD88 inhibitor treatment suggests that IOE (0.2 mg/mL) has equivalent efficacy with that of 10 μM of MyD88 inhibitor on LPS-induced inflammation (Figure 10). These results strongly suggest that IOE could be developed as a potential anti-inflammatory agent capable of attenuating LPS-mediated neuroinflammation in the brain.

## 4. Discussion

**Figure 11:**
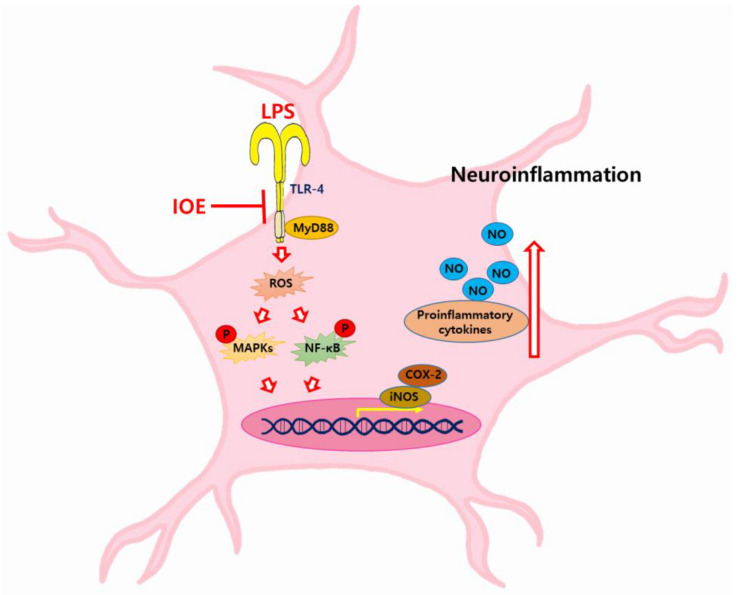
Schematic diagram of the anti-neuroinflammatory effects of IOE. TLR-4/MyD88 mediated inflammatory signaling in brain tissue has been considered as a major pathway to transmit the neuroinflammatory signals. LPS can activate the TLR-4/MyD88 in brain, and it make ROS over-production, lead to MAPKs and NFκB activation. These TLR-4 mediated inflammatory responses stimulate the expression of inflammation mediators such as iNOS, COX-2, proinflammatory cytokines and NO. These continuous responses finally cause neuroinflammation in the brain. Our results suggest that oral administration of IOE can ameliorate the LPS-induced neuroinflammation through attenuating the TLR/MyD88 activation and its downstream inflammatory responses.

Neuroinflammation is known to be involved in the pathology of neurodegenerative diseases, such as AD, Parkinson’s disease, and amyotrophic lateral sclerosis [5]. Inflammatory responses could be stimulated by endogenous factors, such as protein aggregation and genetic mutation, or by environmental causes, such as infection and drug [26,27]. AD-related inflammatory responses could occur due to various sources of infection, such as viruses, fungi, and bacteria [28]. Further, microbiome dysbiosis is considered a contributor to AD pathogenesis [29,30]. Since the infection of gram-negative bacteria is known to cause systemic inflammation and neuronal loss with cognitive impairments, strategies for suppressing inflammatory responses by LPS are receiving much attention in the treatment of neurodegenerative diseases. Thus, in this study, we focused on evaluating the preventive roles of IOE on LPS-induced neuroinflammatory progress using an ICV-injected LPS mouse model and C6 glioma cells. We found that the oral administration of IOE attenuated LPS-induced cognitive impairment and neuronal loss. Furthermore, LPS could not increase the expression of inflammatory mediators, such as COX-2 and iNOS, in the brains of mice fed with IOE as well as C6 glioma cells treated with IOE. These results suggest that IOE effectively ameliorated neuroinflammatory responses due to LPS-induced neurodegenerative progress (Figure 11).

TLRs are pattern recognition receptors (PRPs) that can recognize a common feature of pathogens and play a critical role in the innate immune system. TLR-4 is known to specifically react with LPS and is implicated in cognitive deficits. Studies have shown evidence about involvement of TLR-4 in AD disease that the postmortem brain of AD patients exhibited high levels of TLR-4 expression, and once amyloid precursor protein (APP) is overexpressed in mouse brains, the upregulation of TLR-4 expression was detected [31]. Moreover, cognitive deficits induced by Aβ peptide injection were abolished in TLR-4 knockout mice [32]. These reports strongly suggest that TLR-4 could be a potential therapeutic target for AD progress. Interestingly, we found that the LPS-induced TLR-4 mediated inflammatory signaling pathway was abolished by oral administration of IOE through inactivating the TLR-4 mediated signaling cascade. The expression of TLR-4 and its adaptor protein MyD88 induced by LPS was dramatically attenuated in the brains of the IOE-treated mouse group, indicating that IOE could be used as functional materials that are able to prevent TLR-4-mediated activation when neuroinflammation occurs by infection with gram-negative bacteria.

Infectious agents that can induce neuroinflammation in the brain are recognized by the central system (CNS) and invoke immunological responses. Astrocytes are a major population of glia cells in the CNS and are known to be important regulators of immune responses when the CNS is damaged [33,34]. In addition, TLR-4-mediated signaling pathways in astrocytes have been reported as crucial regulators of neuroinflammation [35]. Furthermore, astrocytes play an important role in up-taking and degrading Aβ in brain [36]. Therefore, we further confirmed the anti-neuroinflammatory effects of IOE on C6 glioma cells, which is a representative astrocyte culture models [37,38]. LPS-induced activation of TLR-4 signaling, which was detected in mouse brains, was also shown in C6 glioma cells, and those inflammatory responses were abolished by IOE treatment in C6 glioma cells. We found that the formation of Aβ plaque in mouse brains was increased by intracerebroventricular LPS injection, but it was significantly decreased by IOE administration. Although the detailed molecular mechanisms on how the Aβ plaque is increased by LPS administration are still unclear, the reduced activity of Aβ clearance by astrocytes may be a cause of Aβ plaque increase in the brain of ICV LPS-injected mice [36]. Therefore, these results suggest that TLR-4-mediated inflammatory signaling pathways in astrocytes could be a target of the anti-neuroinflammatory effects of IOE.

Oxidative stress, including ROS generation in astrocytes, has been recognized as a crucial mediator in LPS-induced neuroinflammation [16,39]. TLR-4 activation induced by LPS stimulation is closely connected with ROS overproduction to control proinflammatory cytokines and NO production in various inflammatory responses [40,41]. In this study, we demonstrated that IOE inhibited LPS-induced TLR-4 activation and ROS overproduction in C6 glioma cells. ROS overproduction induced by LPS treatment was remarkably reduced by NBP2–29328, an inhibitor of MyD88, providing direct evidence that TLR-4 activation is connected with ROS production in LPS-treated C6 glioma cells. 

TLR-4-dependent MAPKs and NFκB signaling pathways in inflammation have received much attention because they play important regulatory roles in the expression of proinflammatory cytokines and the production of NO triggered by LPS [35,41]. Furthermore, attenuating LPS-mediated ROS generation seems to be an upstream position to activate the MAPKs and NFκB in the inflammatory response [42,43]. We showed that the reduction in LPS-induced phosphorylation of MAPKs and NFκB on the IOE-treated mouse brains and C6 glioma cells indicates the involvement of MAPKs and NFκB signaling pathways in IOE-mediated anti-neuroinflammatory activity. In addition, since the overproduction of ROS triggered by LPS was ameliorated by IOE treatment in C6 glioma cells, our data suggest that IOE may target the TLR-4/MyD88/ROS/MAPKs or TLR-4/MyD88/ROS/NFκB pathways to prevent LPS-induced neuroinflammation (Figure 11).

MyD88 is a well-known adaptor protein of TLRs that regulates their signal transduction, and numerous studies have shown the importance of MyD88-dependent signaling in TLR function. Actually, mice in which MyD88 is genetically inactivated exhibited no responses to the TLR-4 ligand LPS [44], TRL-2 ligand peptidoglycan [45], TLR-9 ligand CpG DNA [46], and TLR-5 ligand flagellin [47] indicate that MyD88 is an essential component for activating most TLR family members. Therefore, MyD88 has been implicated as a molecular target to cure the TLRs mediated disease progresses [48,49,50]. In this study, we also found that a MyD88 inhibitor (NBP2–29328) could ameliorate the LPS-induced expression of inflammatory mediators in C6 glioma cells. In addition, IOE attenuated the LPS-induced expression of MyD88 in mouse brains as well as C6 gliomas. Furthermore, IOE (0.2 mg/mL) has similar efficacy to 10 μM of MyD88 inhibitor (NBP2–29328) in recovering the LPS-mediated abnormal expression of inflammatory mediators in C6 glioma cells. These results suggest that IOE could be used as a strong MyD88 blocker capable of preventing LPS-induced neuroinflammation.

Several single components of IOE such as diphlorethohydroxycarmalol (DPHC) and isoploroglucin A (IPA) have been isolated and their anti-hyperglycemia activity [51], protective effect of inflammatory myopathy [52] and anti-obesity activity [53] have been demonstrated. Since anti-neuroinflammatory activity of IOE was firstly evaluated in this study, identification of functional single compounds in IOE with anti-neuroinflammatory properties will be performed in our next study.

## 5. Conclusions

In this study, the effects of IOE against LPS-induced neuroinflammation were evaluated for the first time. Oral administration of IOE effectively ameliorated LPS-mediated neuronal loss and cognitive impairments. We also found that IOE could effectively attenuate the LPS-induced activation of TLR-4/MyD88 dependent signaling pathways, resulting in reduced expression of inflammatory mediators in mouse brains and C6 glioma cells. Taken together, these results strongly suggest that IOE could be developed as a potential anti-neuroinflammatory agent that is able to regulate the LPS-induced TLR-4/MyD88 dependent signaling pathways.

## Data Availability

The data used in this study are available from the corresponding author upon request.
